# Dementia knowledge among caregivers with and without training in Lima-Peru

**DOI:** 10.1590/1980-5764-DN-2024-0270

**Published:** 2025-12-01

**Authors:** Belen Custodio, Rosa Montesinos, Marco Malaga, Diego Chambergo-Michilot, Rossana Cruz del Castillo, Graciet Verastegui, Katherine Agüero, Zadith Yauri, Nilton Custodio

**Affiliations:** 1 Instituto Peruano de Neurociencias, Research Department, Lima, Peru.; 2 Equilibria, Research Department, Lima, Peru.; 3 Universidad de San Martín de Porres, Facultad de Medicina, Centro de Investigación del Envejecimiento, Lima, Peru.; 4 Universidad Científica del Sur, Lima, Peru.

**Keywords:** Dementia, Caregivers, Health Education, Burnout, Psychological, Latin America, Demencia, Cuidadores, Educación en salud, Agotamiento Psicológico, América Latina

## Abstract

Dementia prevalence is increasing globally, especially in low- and middle-income countries like Peru. Evidence has shown that a trained caregiver improves the patient’s quality of life, reduces behavioral symptoms, and decreases the likelihood of the caregiver developing burnout.

**Objective:**

The aim of the study was to measure the level of knowledge of caregivers attending monthly training compared to untrained caregivers in Lima, Peru.

**Methods:**

A cross-sectional study was conducted involving 102 informal caregivers of dementia patients, split evenly into trained (n=51) and untrained (n=51) groups. The training program ("Lonchecito para cuidadores") consists of 10 virtual meetings per year, where the main topics are: types of dementia, management of behavioral symptoms, application of non-pharmacological measures, and caregiver well-being. Dementia knowledge was assessed using the Spanish version of the Dementia Knowledge Assessment Scale (DKAS-S). Data were analyzed using Student’s t-test to compare group scores, with a significance threshold of p<0.05.

**Results:**

The trained caregivers scored higher [mean=18, standard deviation (SD)=2.72] than the untrained group (mean=16, SD=2.6) (p=0.001). Higher education was moderately correlated with better DKAS-S scores. Notably, questions on early dementia symptoms, advanced care, and management of behavioral symptoms had the highest error rates, with up to 70% incorrect responses on some items.

**Conclusion:**

Caregivers with dementia training performed better than the general population, underscoring the need for improved community access to quality dementia information.

## RESUMEN.

La prevalencia de demencia está aumentando a nivel mundial, especialmente en países de ingresos bajos y medianos como Perú. La evidencia ha demostrado que un cuidador capacitado tiene un impacto positivo en la calidad de vida del paciente, reduce los síntomas conductuales y disminuye la probabilidad de que el cuidador desarrolle burnout.

**Objetivo:**

Medir el nivel de conocimiento de cuidadores que asisten a capacitaciones mensuales en comparación con cuidadores no capacitados en Lima, Perú.

**Métodos:**

Se realizó un estudio transversal con 102 cuidadores informales de personas con demencia, distribuidos equitativamente en un grupo capacitado (n=51) y un grupo no capacitado (n=51). El programa de capacitación (“Lonchecito para cuidadores”) consiste en 10 reuniones virtuales al año, donde los principales temas son: tipos de demencia, manejo de síntomas conductuales, aplicación de medidas no farmacológicas y bienestar del cuidador. El conocimiento sobre demencia se evaluó mediante la versión en español de la Dementia Knowledge Assessment Scale (DKAS-S). Los datos se analizaron utilizando la prueba T de Student para comparar los puntajes entre grupos, con un umbral de significancia de p<0,05.

**Resultados:**

Los cuidadores entrenados obtuvieron puntajes más altos (media=18, DE=2.72) que el grupo no entrenado (media=16, DE=2.6) (p=0.001). Un mayor nivel educativo se correlacionó de manera moderada con mejores puntajes en la DKAS-S. Cabe destacar que las preguntas sobre síntomas tempranos de la demencia, cuidados avanzados y manejo de síntomas conductuales presentaron las tasas más altas de error, con hasta un 70% de respuestas incorrectas.

**Conclusión:**

Los cuidadores con capacitación en demencia tuvieron un mejor desempeño que la población general, lo que resalta la necesidad de ampliar el acceso comunitario a información de calidad sobre demencia.

## INTRODUCTION

 As global life expectancy continues to rise, the proportion of older adults has grown substantially, accompanied by an increase in degenerative conditions such as dementia^1^. In 2021, approximately 57 million people were living with dementia worldwide, with nearly 10 million new cases diagnosed annually^2,3^, and projections indicate that this number could exceed 150 million by 2050^1^. The burden is particularly high in low- and middle-income countries, which accounted for over 60% of all cases in 2021. In Latin America (LA), prevalence is estimated at 9–10%, with more than 20 million new cases expected by 2050^4^. In Peru, dementia cases are projected to rise by 279%, from 196,699 in 2019 to approximately 744,847 by 2050^1^. 

 Given this alarming outlook, dementia has been recognized as a public health priority due to its profound economic, social, and health impact^5^. In response, the World Health Organization (WHO) launched the Global Action Plan on the Public Health Response to Dementia 2017–2025, which prioritizes dementia through seven strategic areas, including awareness, risk reduction, diagnosis, treatment, caregiver support, information systems, and research and innovation^6^. 

 Awareness and understanding of dementia are essential for early detection and for combating the stigma surrounding the disease^7^. Additionally, evidence shows that trained caregivers, those familiar with the pathology, disease progression, and appropriate responses to symptoms, can improve patients’ quality of life, reduce behavioral and psychological symptoms of dementia (BPSD), and lower the risk of caregiver burnout^8,9^. 

 Several international initiatives have been developed to provide educational support for dementia caregivers^10^. Among them, the WHO’s iSupport program is a self-guided, online training designed to improve caregivers’ knowledge, coping skills, and self-care practices, and has been adapted in multiple languages^11^. Other interventions, such as the Savvy Caregiver Program^12^ and the Resources for Enhancing Alzheimer’s Caregiver Health intervention^13,14^, have been implemented mainly in high-income countries. In Peru, however, Lonchecito para Cuidadores is, to our knowledge, the only structured, fully online training program for dementia caregivers. Delivered through virtual workshops, it addresses key topics with content culturally adapted to the Peruvian context. Unlike iSupport, which is primarily self-administered, Lonchecito para Cuidadores is delivered in real time by multidisciplinary professionals, enabling interactive participation, personalized feedback, and peer support. 

 Caregivers in LA are frequently family members of the person with dementia. In Peru, 81.5% are women, and 60.87% are spouses of the patient^15^. In addition, caregivers in Peru have reported reduced time for employment, personal activities, and perceived declines in health^15^. Despite their central role, research on dementia knowledge among caregivers remains scarce in the region, with most studies focusing instead on healthcare professionals, students, or the general public^16^. Moreover, even in countries where such studies exist, there is little standardization in measurement tools, and many researchers develop their own instruments despite the availability of validated scales^8^. 

 For this study, two validated tools were considered: the Alzheimer’s Disease Knowledge Scale (ADKS) and the Dementia Knowledge Assessment Scale (DKAS). While both have been validated for use with caregivers^17-19^, the DKAS demonstrated superior internal consistency and better discrimination to pre–post training changes^20^. Given these advantages and its prior translation and validation in Spanish-speaking populations^5^, the DKAS was selected. 

 This study aimed to compare the level of knowledge of dementia caregivers who attend a training program to those who do not receive any training in Lima, Peru, using a validated questionnaire. 

## METHODS

### Study design and setting

 A cross-sectional study was designed to assess the level of knowledge of trained and untrained dementia caregivers. The study was conducted in the Research Department of the Instituto Peruano de Neurociencias—IPN, a neurology clinic in Lima-Peru, between December 2023 and June 2024. 

### Participants

 Participants were recruited using a convenience sampling method of adults declared as caregivers of patients with dementia. Trained caregivers were recruited within the training program "Lonchecito para Cuidadores." Untrained caregivers were recruited by presenting the study to caregivers of patients receiving treatment at the IPN who were not part of the training program. Inclusion criteria were:

- Being ≥18 years old;
- Residing in Lima, Peru;
- Providing care to a person with dementia for a minimum of 6 h per day at least 3 days per week;
- Giving written informed consent to participate in the study. Caregivers who did not meet these criteria were excluded.

This study was approved by the Ethics Board of the Hospital Nacional Docente Madre Niño "San Bartolomé" in Lima-Peru. 

### Training program

 "Lonchecito para Cuidadores" is a structured training program for dementia caregivers, consisting of 10 monthly virtual meetings delivered between February and November via Zoom. The curriculum was designed collaboratively by a multidisciplinary team including neurologists, physiatrists, geriatricians, psychiatrists, and psychologists specialized in cognitive training, and it was informed by evidence-based dementia care guidelines, caregiver training needs identified in preliminary surveys, and local cultural considerations. 

**Figure 1:**
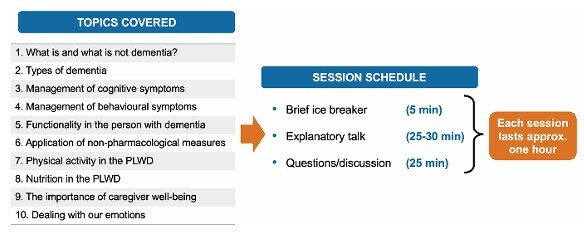
Training program structure.

 The sequence of topics (Figure 1) was organized to progress from general knowledge of dementia to practical caregiving strategies and caregiver well-being. Each session followed a participatory model combining brief rapport-building activities, interactive lectures, and open discussion. 

 During implementation, facilitators included the accessibility of the online format, the cultural adaptation of materials to the Peruvian context, and the opportunity for real-time interaction with experienced professionals. Challenges included occasional internet connectivity issues, scheduling conflicts due to caregiving demands, and variable levels of digital literacy among participants. To address these, the team provided technical support before sessions, shared recordings for those unable to attend live, and used simple, visually engaging materials to enhance comprehension. 

### Sample size

 For the sample size calculation, a 95% confidence level (Z-score=1.96) was considered, with an approximate prevalence of 7%, according to a prevalence study in Lima, and a margin of 0.10 (0.05 on each side). Therefore, a total of 102 caregivers were included in the study, 51 trained caregivers and 51 untrained. 

### Instruments

 Data were collected using the DKAS, a validated questionnaire with 25 statements grouped into the following categories: causes and characteristics of dementia, necessary care of a patient with dementia, communication and behavior of a patient with dementia, and risks and promotion of brain health; where the participant must select true or false for each of the questions^18,19^. For this study, the validated Spanish version was used^5^, and the research team reviewed it and made three cultural adaptations to questions 5, 15, and 25, without affecting their initial purpose. Subsequently, 20 people from different socioeconomic and educational levels were consulted to see if all the statements were understandable; based on their comments, two additional modifications were made in questions 3 and 21. Table 1 contains the questionnaire used. Sociodemographic questions were added to the initial questionnaire and were self-reported by participants. 

**Table 1:** DKAS-S, after cultural adaptations (English version).

#	Statement	T	F
1.	Dementia is a normal phase of aging.		
2.	Alzheimer’s disease is the most common form of dementia.		
3.	People with dementia can recover from this disease.		
4.	Dementia is not the result of physical changes in the brain.		
5.	Planning care in the severe/final stage of the disease is generally not necessary.		
6.	Vascular dementia is the most common form of dementia.		
7.	In general, most dementias do not shorten a person’s life expectancy.		
8.	Having high blood pressure increases the risk of developing dementia.		
9.	Maintaining a healthy lifestyle does not reduce the risk of the most common forms of dementia.		
10.	Symptoms of depression can be mistaken for symptoms of dementia.		
11.	Physical exercise is generally beneficial for people with dementia.		
12.	Early diagnosis of dementia generally does not improve the quality of life of patients with the disease.		
13.	The sudden onset of cognitive problems is typical of the most common forms of dementia.		
14.	It is impossible to communicate with a person who has advanced dementia.		
15.	A person with advanced dementia generally does not respond to changes in their usual environment.		
16.	It is important to correct a person with dementia when they are confused.		
17.	People with advanced dementia generally communicate through body language.		
18.	Abnormal behaviors in people with dementia are generally a response to unmet needs.		
19.	Medication is the most effective way to treat the behavioral symptoms of dementia.		
20.	People with dementia generally have no problems making decisions.		
21.	In the later stages of dementia, mobility is affected.		
22.	People with advanced dementia may have difficulty speaking.		
23.	People with dementia often have difficulty acquiring new skills.		
24.	Difficulties with eating and drinking generally appear in the later stages of dementia.		
25.	Daily care of a person with advanced dementia is effective when it focuses on the patient’s well-being.		

### Data analysis

 The characteristics of caregivers were described using frequency measures. Mean scores of the DKAS-S between groups were then compared using a Studen’’s t-test. The number of correct answers, gender, and level of education were correlated using Pearson’s correlation coefficient. Attendance records were not considered in adjusting the analysis. Statistical analysis was conducted using STATA v18. The significance level was set at p<0.05. 

## RESULTS

 The demographic characteristics of all the participants (n=102) are shown in Table 2. Female participation was significantly higher overall, accounting for 78% of the total number of participants. The two largest age groups were 41–50 and 51–60 years. Regarding educational level, most participants reported having a higher education, and the most predominant socioeconomic position (SEP) was medium. Finally, 90% of the caregivers enrolled said that they were informal caregivers. 

**Table 2:** Socio-demographic characteristics of caregivers according to online training.

Characteristics		Part of the training group			
		YES (n=51)		NO (n=51)	
Gender	Female (%)	46	(90.2)	33	(65.0)
	Male (%)	5	(9.8)	18	(35.0)
Age, years	18–30	7	(13.7)	3	(5.8)
	31–40	8	(15.7)	4	(7.8)
	41–50	12	(23.5)	16	(31.4)
	51–60	15	(29.4)	14	(27.5)
	61–70	6	(11.8)	8	(15.7)
	70 +	3	(5.8)	6	(11.8)
Education*	Elementary school	1	(1.9)	1	(1.9)
	High school	6	(11.8)	13	(25.5)
	Higher education—Technical	15	(29.4)	16	(31.4)
	Higher education—College	26	(51.1)	17	(33.3)
	Master’s degree	3	(5.8)	3	(5.8)
	PhD+	0	(0.0)	1	(1.9)
Socioeconomic position—SEP §	Low	12	(23.5)	7	(13.7)
	Medium	38	(74.5)	44	(86.3)
	High	1	(1.9)	0	(0.0)
Caregiver type ||	Formal	7	(13.7)	3	(5.8)
	Informal	44	(86.3)	48	(94.2)

Abbreviation: +PhD, people who have reached a professional degree for a Doctor of Philosophy.

Notes: *Participants selected their highest completed level. In Peru, primary education (“elementary school”) consists of 6 years, followed by 5 years of secondary education (“high school”). The difference between technical education and college is that the former lasts 3 years and focuses on operational roles (equivalent to a “technical degree” or “associate degree” in the United States), while college is provided at a university and lasts 5 years (equivalent to a “bachelor’s degree” in the United States). §Socioeconomic position: determined by family income; || Caregiver type: caregivers were classified as informal if they were family members providing unpaid care or formal if they were hired and compensated for caregiving.

**Figure 2:**
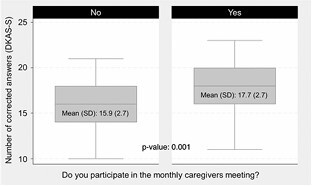
Comparison of knowledge between caregivers with and without training.

 When comparing the level of knowledge between both groups of caregivers (Figure 2), it was found that the mean score for caregivers who received training was 18 [standard deviation (SD)=2.72], while those who did not receive any training had a score of 16 (SD=2.6). The Student’s t-test revealed a significant difference with a p-value: p=0.001. Correlation analysis between the number of correct answers and the variables gender and education level revealed a moderate positive relationship (0.3) between education level and the number of correctly answered questions, suggesting that higher education might be associated with better performance on the DKAS-S. In contrast, the correlations between the number of correct answers and gender, as well as between gender and education, were weak, with coefficients of -0.1 and 0.07, respectively. Additionally, a linear regression analysis was conducted with the DKAS-S score as the dependent variable, the training group as the primary independent variable, and education as a covariate. The results indicated that training was significantly associated with higher DKAS-S scores (β=1.61, p=0.002), even after controlling for education level (β=0.77, p=0.005). This finding suggests that the positive effect of training on DKAS-S performance is independent of participants’ educational backgrounds. 

 A relevant finding is that 72% or more of the caregivers answered questions 13, 15, and 19 incorrectly, which are related to the onset symptoms of dementia, care in advanced stages, and management of behavioral symptoms, respectively. Question 13 had the worst performance, with 90% (92 participants) of the total sample answering incorrectly. All DKAS-S responses can be found in Table 3. 

**Table 3:** DKAS-S answers per question.

Statement content	# of correct answers	# of incorrect answers
1. Most forms of dementia do not generally shorten a person’s life	56	46
2. Blood vessel disease (vascular dementia) is the most common form of dementia	75	27
3. People can recover from the most common forms of dementia	90	12
4. Dementia is a normal part of the ageing process	76	26
5. Dementia does not result from physical changes in the brain	89	13
6. Planning for end-of-life care is generally not necessary following a diagnosis of dementia	51	51
7. Alzheimer’s disease is the most common form of dementia	50	52
8. It is impossible to communicate with a person who has advanced dementia	56	46
9. A person experiencing advanced dementia will not generally respond to changes in their physical environment	65	37
10. It is important to correct a person with dementia when they are confused	79	23
11. People experiencing advanced dementia often communicate through body language	97	5
12. Uncharacteristic behaviors in a person experiencing dementia are generally a response to unmet needs	78	24
13. Medications are the most effective way of treating behavioral symptoms of dementia	10	92
14. People experiencing dementia do not generally have problems making decisions	52	50
15. Movement is generally affected in the later stages of dementia	28	74
16. Difficulty eating and drinking generally occurs in the later stages of dementia	66	36
17. People with advanced dementia may have difficulty speaking	66	36
18. People experiencing dementia often have difficulty learning new skills	56	46
19. Daily care for a person with advanced dementia is effective when it focuses on providing comfort	24	78
20. Having high blood pressure increases a person’s risk of developing dementia	87	15
21. Maintaining a healthy lifestyle does not reduce the risk of developing the most common forms of dementia	92	10
22. Symptoms of depression can be mistaken for symptoms of dementia	94	8
23. The sudden onset of cognitive problems is characteristic of common forms of dementia	89	13
24. Exercise is generally beneficial for people experiencing dementia	88	14
25. Early diagnosis of dementia does not generally improve the quality of life for people experiencing the condition	100	2

## DISCUSSION

 This pilot study (Lonchecito para Cuidadores) is the first in Peru to assess the feasibility of an online training program to improve dementia knowledge in carers using the DKAS-S, finding higher scores among regular attendees. 

 The use of online health interventions has increased in the last few years, especially in low- and middle-income countries (LMIC)^21^, and the development of accessible, acceptable, and effective caregiver support programs is a strategic priority in the global dementia action plan^6^. Online interventions can increase adherence by eliminating the need to commute and allowing integration into the daily care routine^22,23^. Studies also report benefits such as reduced anxiety, depression, and stress, as well as improved well-being and quality of life^24-26^. 

 Dementia carers have reported needing information, support with symptom management, their own health, and social balance^27^. Especially in lower-resourced settings, such as LA, a huge gap exists whereby there are not enough care providers, and yet, there is a rapidly growing rate of dementia. Given this, the training and support for family and other caregivers to care for dementia patients is important. Various initiatives are attempting to address this gap; for example, the WHO developed “iSupport,” an evidence-informed e-health intervention designed to help dementia carers provide good care and take care of themselves, which is in the process of adaptation and validated in several countries in Europe, Asia, Oceania, and Brazil in LA^28^. 

 A lack of standardization in the use of tools to measure the level of knowledge in dementias has been reported^8^, as evidenced by Farina et al.^29^ in their systematic review where they sought to compile all the experiences in LA but only identified four articles in Brazil measuring different aspects of knowledge in dementias, and only one article used a previously validated tool; the other three studies used questionnaires created for the study based on similar studies, and not all the questions were related to knowledge. Hence, this study, to our knowledge, is the first in Peru and one of the few in LA to use a validated scale to measure the level of knowledge of dementia in caregivers. In Brazil, the study, which used a validated instrument, applied the ADKS, reporting an average score of 21.59 (22/30); however, when health personnel who represented 36% of the sample were excluded, the score decreased to 20.5. Overall, the study demonstrated that those with more years of education performed better^30^. On the other hand, the remaining studies created their own questionnaires to measure knowledge about Alzheimer’s disease (AD) or dementias. Matioli et al.^31^ conducted a study in Santos-Brazil, to assess knowledge of AD in an educated population of older adults, where the association between higher knowledge and higher educational level was also found. This was measured through a questionnaire with sociodemographic and knowledge yes/no questions, where more than half of the respondents answered that memory loss is normal for aging and 3/4 of the sample had never visited a doctor to evaluate their memory. Similarly, another study in Botucatu-Brazil, also identified that only a minimal percentage of their sample would consider seeking medical help to assess their memory^32^. Similar to what was reported by Matioli et al.^31^. In Peru, the level of knowledge of dementia among health professionals has also been reported; however, the instrument used had not been previously validated either. The study identified that the healthcare professionals had a low level of knowledge about dementias in general and specifically about AD, with more than half of the sample identifying ‘senile dementia’ as a valid type of dementia^33^. 

 A Portuguese study identified "care interactions", such as managing BPSD and communicating with people with dementia, as the main training needs^34^, aligning with our findings on advanced care and behavioral symptom management. In contrast, the Brazilian study that used the ADKS found high accuracy in domains like symptoms and diagnosis, whereas our participants showed a 90% error rate regarding the onset symptoms of dementia. The low accuracy observed in questions related to early symptoms, advanced stage care, and the management of behavioral symptoms has important implications for the patients and caregivers. Limited knowledge of the early signs of dementia can delay diagnosis and access to timely support^7^, while inadequate understanding of behavioral and psychological symptoms may lead to ineffective care strategies and negative mental health outcomes for the caregiver^8,9^. These findings highlight the need to strengthen these topics within caregiver training programs. 

 Our study supports the evidence for the efficacy of online training in dementia knowledge for caregivers compared to those who did not receive any training. The advantage observed among caregivers who received training compared to the general population may be explained by the multidimensional nature of the training program, which combined theoretical content on dementia, practical strategies for symptom management, and self-care techniques for the caregivers themselves. This combination contributed to both an increase in disease-specific knowledge and a strengthened sense of competence. As highlighted by González-Fraile et al.^35^ in their systematic review, educational interventions aim to improve caregivers’ knowledge, enhance their sense of competence, and strengthen their ability to cope with challenging situations. Additionally, as suggested in the adult education literature, factors such as content relevance, practical applicability, and perceived self-efficacy can facilitate learning and the retention of new knowledge^36,37^. In our program, key components appeared to be: synchronous sessions focused on everyday caregiving challenges (e.g., nighttime wandering, agitation) and culturally adapted, easy-to-understand materials that avoided excessive medical jargon. The combination of relevant content, accessible language, and interactive pedagogical methods created a more robust learning environment than what is typically available to the general population, which may explain the higher performance observed among trained caregivers and supports the value of online psychoeducational interventions to improve dementia knowledge. 

 Nevertheless, evidence for technology-based counseling remains inconclusive. A recent meta-analysis, including five randomized controlled trials, found no significant pooled effects on depressive symptoms, burden, and self-efficacy/mastery, although individual studies reported some beneficial effects on outcomes, such as caregiver reaction to dementia-related behavior and service utilization^38^. These mixed findings highlight that online counseling and online education are distinct modalities addressing different caregiver needs. Our results add specific evidence that targeted, culturally sensitive e-learning can improve dementia knowledge, an essential foundation upon which future technology-assisted counselling interventions may build to achieve broader psychosocial gains, and they also highlight the need to expand access to quality dementia information within the community, for example, through brain health campaigns, free online modules with relevant content, and, above all, by providing continuous refresher training for caregivers of people with dementia, as this has a positive impact on both the person with dementia and their caregiver. 

### Strengths and limitations

 This study addresses important gaps in the literature on the training needs of dementia caregivers in Lima, Peru. Its main strengths include: addressing a socially relevant and pressing public health issue by generating locally applicable evidence in a population with a high caregiving burden and for which no previous data were available; and the use of the validated DKAS-S questionnaire, setting a precedent for future regional and national research. However, several limitations should be noted. The observational design, lack of group randomization, and convenience sampling may have introduced selection bias. Less resourceful caregivers, who may have lacked the time or motivation to participate, are likely underrepresented. Another important limitation is that participants were assessed only once; a pre–post evaluation would have allowed a more robust assessment of the impact of the training. The high educational level of participants (86.3% in the training group and 70.5% in the control group with ≥12 years of education, compared to 43.7% in the general Peruvian population) may reflect the higher educational attainment in Lima and the reliance on online questionnaires, which tend to attract more highly educated individuals. The sample was predominantly female, with particularly low male participation (9.8% in the training group and 35% in the control group). Finally, all participants resided in Lima, Peru, limiting the generalizability of the findings to caregivers from other regions or rural settings. Future work should aim to include a more gender-balanced sample, broaden recruitment to caregivers from diverse regions of the country, and incorporate pre–post assessments to better evaluate training effectiveness. 

 In conclusion, our study found that trained caregivers demonstrated higher knowledge levels regarding dementia compared to untrained caregivers, and knowledge was also positively correlated with education level. These results suggest that virtual education programs may effectively enhance dementia knowledge among informal caregivers. However, further studies with improved sampling methods are necessary to confirm these findings and minimize potential biases. Addressing dementia knowledge gaps through targeted, accessible training could play a crucial role in better supporting caregivers in LA. 

## Data Availability

The authors are willing to allow the journal to review their data if requested.
